# Disability rights and experiential use of psychedelics in clinical research and practice

**DOI:** 10.1038/s44184-024-00065-y

**Published:** 2024-07-02

**Authors:** Maryam Golafshani, Daniel Z. Buchman, M. Ishrat Husain

**Affiliations:** 1 MD Program, Temerty Faculty of Medicine, University of Toronto, Toronto, ON Canada; 2 Campbell Family Mental Health Research Institute, Centre for Addiction and Mental Health, Toronto, ON Canada; 3 Dalla Lana School of Public Health, University of Toronto, Toronto, ON Canada; 4 Department of Psychiatry, Temerty Faculty of Medicine, University of Toronto, Toronto, ON Canada

**Keywords:** Psychiatric disorders, Medical ethics

## Abstract

Given the renewed interest in the use of psychedelics for the treatment of mental and substance use disorders in recent decades, there has also been renewed discussion and debate about whether it is necessary or beneficial for those who study and deliver psychedelic-assisted psychotherapy (PAP) to have had personal experience of using psychedelics. This paper provides a brief history of this debate and brings a disability-rights perspective to the discussion, given increasing efforts to dismantle ableism in medical training, practice, and research. Many psychiatric conditions and psychotropic medications, including ones as commonly prescribed as antidepressants, may preclude one from being able to safely and/or effectively use psychedelics. As such, we argue explicitly mandating or even implying the necessity of experiential training for psychedelic researchers and clinicians can perpetuate ableism in medicine by excluding those who cannot safely use psychedelics because of their personal medical histories. As PAP research and practice rapidly grow, we must ensure the field grows with disability inclusion amongst researchers and clinicians.

With renewed interest in the use of psychedelics for the treatment of mental and substance use disorders over the last two decades^1^, there has also been renewed discussion and debate about whether it is necessary for those who study and deliver psychedelic therapies to have had personal experience of using psychedelics. At the same time, there has been significant recognition of and work toward dismantling ableism in medical training, practice, and research. In this commentary, we add a disability rights perspective to the debate about the experiential use of psychedelics—a consideration that has been overlooked in our knowledge. We argue that explicitly mandating or even implying the necessity of experiential training for psychedelic researchers and clinicians can perpetuate ableism in medicine by excluding those who cannot safely use psychedelics because of their personal medical histories.

The question of whether personal psychedelic experience is beneficial or necessary for psychedelic research and clinical practice dates back to the initial development and use of medicalized psychedelics in the 1950s and 1960s. During this initial era of psychedelic research, it was argued that first-hand experience made it easier for researchers and clinicians to adequately understand, interpret, and guide patients through psychedelic-assisted psychotherapy (PAP)^2^. When psychedelics were reclassified as Schedule 1 narcotics leading to an abrupt cessation of psychedelic research in the early 1970s, the vast majority of knowledge, opinion, and practice of psychedelic therapy was formed by non-medical professionals—often dubbed psychedelic “guides”—in an unofficial, illicit capacity that was often anti-establishment and counter-cultural. Psychedelic guides are often led to the work through their own positive experiences of using psychedelics and tend to hold strong beliefs that one can only support someone else through a psychedelic experience if they have had their own. Given this history of psychedelic use and that psychedelics are indeed still primarily used in non-regulated settings, there persists a culture and impression that first-hand psychedelic experience is, if not definitively necessary, at least greatly beneficial to researchers and clinicians working in regulated settings.

Today, first-hand psychedelic experience may include regulated PAP in a clinical or research context, as well as unregulated psychedelic use in non-clinical contexts (e.g., for non-medical purposes or with non-licensed guides). While there is limited formal data, there is a widely held assumption that most psychedelic researchers and clinicians have used psychedelics in regulated or unregulated settings^3,4^. Indeed, there is precedent for the former type of use: many psychotherapeutic training programs—from psychoanalysis to arts-based therapy—require learners to simultaneously undergo the same therapy they are learning to provide others. To our knowledge, there are no official requirements for experiential training for psychedelic researchers and clinicians in regulated medical settings. Nonetheless, many training guidelines recommend experiential training, and so the debate on the necessity of experiential training persists^5,6^.

With psychedelics re-entering the fold of institutionally-regulated, evidence-based psychiatric research and practice, there is renewed interest in whether first-hand psychedelic experience is, in the first instance, beneficial and then actually necessary^2–4,7^. The debate has centered largely, though not exhaustively, around (1) whether personal experience with regulated PAP in a clinical or research context is necessary or beneficial to learn how to effectively deliver psychedelic therapy and (2) whether personal use of psychedelics in both clinical and non-clinical settings may bias researchers’ and clinicians’ critical appraisal of the evidence on the safety and efficacy of psychedelic therapies. Alternatively, whether the lack of (1) personal experience with PAP is detrimental to one’s ability to deliver effective psychedelic therapy and (2) personal use in both clinical and non-clinical contexts may bias researchers and clinicians against psychedelic use in psychiatry. The latter question about bias is increasingly critical in recent decades with the rise of evidence-based medicine, wherein the objectivity of research and its clinical application is deemed to be paramount for the safe and effective practice of medicine. Interestingly, arguments for and against personal psychedelic use are not themselves evidence-based; rather, the discussion is still theoretical and has yet to be empirically studied^2^.

Considering disability in the debate on personal psychedelic use is not just an empirical scientific concern. It is also a normative social justice concern that is a necessary part of growing efforts to advance diversity, equity, and inclusion in medicine. Over the last two decades, there have been increasing efforts to reduce barriers for health professional learners with disabilities, de-stigmatize personal experiences of illness and disability amongst healthcare professionals, and tackle ableism in medicine more generally^8,9^. These efforts matter not only so health professions education meets the basic rights of people with disabilities but also because clinicians with lived experience of disability may better understand the nuanced needs of the growing number of patients with disabilities, and addressing ableism supports a broader structure and culture of wellness in medicine and other health professions. As such efforts continue and increase, there will inevitably be increasing numbers of trainees and clinicians with disabilities, including chronic mental health conditions, that may preclude them from using psychedelics themselves.

Currently, the majority of research and use of psychedelics in psychiatry includes classic serotonergic psychedelics, most commonly psilocybin, for major depressive disorder and treatment-resistant depression^10^. There is also increasing research on the use of the entactogen methylenedioxy-methylamphetamine (MDMA) for posttraumatic stress disorder and social anxiety^11^. Most clinical trials of psychedelic therapies require participants to first have a partial or full washout of psychotropic medications, including antidepressants, antipsychotics, and mood stabilizers, due to potential interactions with the study drugs, such as a theoretically increased risk of serotonin syndrome with serotonergic psychedelics^10,12^. In addition, participants with a personal or family history of psychosis and mania are excluded from most psychedelic trials due to a theoretically increased risk of psychosis from psychedelic use^10^. In clinical practice, the patient and their psychiatrist weigh the benefits versus risks of the psychedelic treatment, given the relative contraindications that are relevant to the patient. Practically, this means many researchers and clinicians who already take psychotropic medications—including ones as common as selective serotonin reuptake inhibitors (SSRIs)—or have certain personal or family psychiatric histories may not be eligible for psychedelic therapies.

Consider the example of depression amongst medical students: a recent meta-analysis found the prevalence of depression and depressive symptoms amongst medical students to be 27.2% across 43 countries, with 15.7% of students who screened positive for depression seeking psychiatric treatment^13^. While the authors do not specify what percentage of these medical students were specifically treated with antidepressants, it is likely that a substantial proportion are prescribed antidepressants, let alone other psychotropic medications. Given how common antidepressant use is likely to be amongst medical students and the aforementioned risk of serotonin syndrome, many medical students may be excluded from psychedelic research and practice if personal experience is considered necessary due to their own mental health, making this a disability justice issue. This ableism would be most obvious if experiential training eventually becomes mandatory, but even an assumption within the field that personal psychedelic experience is necessary and beneficial can be ableist by discouraging those who do not have personal psychedelic experience from pursuing research and practice of psychedelic therapies.

Interestingly, a recent article highlights a similar need for the inclusion of patients with physical and sensory disabilities in psychedelic research and practice because of the substantial mental health burden they face^14^. The authors not only seek to challenge ableism in medicine but also a more general problem with evidence-based medicine: that clinical trial participants, especially in randomized controlled trials, often do not reflect the diversity of patients in real-world clinical practice—in terms of gender, race, socioeconomic status, multimorbidity, and more—to whom such evidence is actually applied. We argue the flip side also matters: that researchers and clinicians who are contributing to and applying evidence-based medicine reflect the diversity of patient populations, especially in an emerging area like PAP where many patients have disabling, treatment-resistant mental disorders.

There is growing consensus and evidence that increasing the diversity of the physician workforce actually improves outcomes for patients, especially underserved and marginalized patient populations like ones with disabilities^15,16^. The same could be argued with regard to diversity amongst researchers who may be more inclined to ask critical research questions relevant to diverse patient populations and also ensure study participants better reflect the diversity of populations. In these ways, lived experience of the systemic challenges diverse patient populations face is beneficial. Here is the irony with the argument for experiential psychedelic use: while it privileges the lived experience of the intervention amongst researchers and clinicians, it can exclude those with lived experience of the mental health challenges and disability that their patients face. If PAP is to become widely accessible through clinical trials and eventually clinical practice, we must be careful it is done so with attention to disability justice and inclusion.
